# Expression of chronic myeloid leukemia oncogenes BCR-ABL ^P210^ and BCR-ABL ^T315I^ affect cellular and humoral innate immunity in *Drosophila melanogaster*

**DOI:** 10.17912/micropub.biology.000551

**Published:** 2022-04-13

**Authors:** Dana Abubaker, Amro Baassiri, Mirna Ghannam, Amani Al Outa, Ali Ghais, Elias Rahal, Rihab Nasr, Margret Shirinian

**Affiliations:** 1 Department of Experimental Pathology and Immunology, Faculty of Medicine, American University of Beirut, Lebanon; 2 Center for Infectious Diseases Research, American University of Beirut Medical Center, Beirut, Lebanon; 3 Department of Anatomy, Cell Biology and Physiological Sciences, Faculty of Medicine, American University of Beirut, Lebanon

## Abstract

Chronic myeloid leukemia (CML) is a myeloproliferative neoplasm that results from a chromosomal translocation between chromosome 9 and chromosome 22. The resulting fusion gene ( *BCR-ABL* ) encodes a constitutively active BCR-ABL tyrosine kinase. Some mutations of this oncogene, especially the Threonine 315 to Isoleucine substitution of the ABL kinase is resistant to first and second-generation tyrosine kinase inhibitors (TKIs) conventionally used in CML therapy. We have previously validated a CML fruit fly model for drug screening using the adult fly compound eye. Here we expressed wild-type *BCR-ABL ^P210^* and mutated *BCR-ABL ^T315I^* in *Drosophila melanogaster* hematopoietic system to understand the phenotypic consequences of this expression and its impact on innate immune pathways. Flies expressing both wild-type *BCR-ABL* ^P210^** and mutant *BCR-ABL* ^T315I^ showed increased number of circulating hemocytes, disruption in sessile patterning of resident hemocytes, dysregulation in the humoral Toll, ImD, and JAK/STAT pathways at the mRNA level in both the 3 ^rd^ instar larva and adult stages. Of note, *BCR-ABL* ^T315I^ flies presented more severe phenotypes and a higher deviation in humoral dysregulation than BCR *-ABL* ^P210^ flies pointing towards more complex oncogenic effect of this mutant which requires further investigation.

**Figure 1. Expression of BCR-ABL P210 and BCR-ABL T315I in the Drosophila melanogaster hematopoietic system results in an increase in hemocyte count, disruption in the patterning of the sessile hemocytes, and TotA overexpression which persists from 3 rd instar larval to adult stage in BCR-ABL T315I flies :**
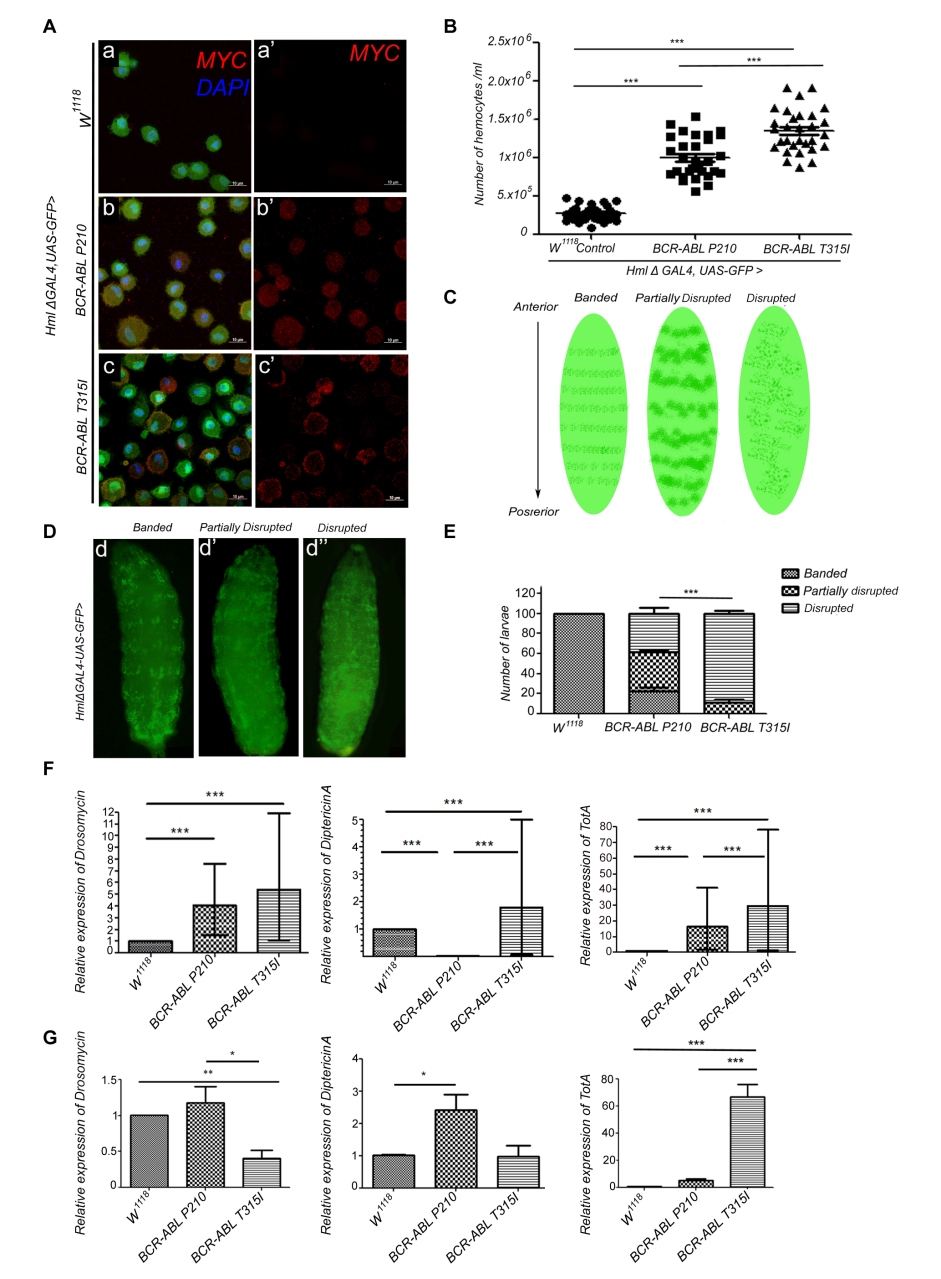
(A) Maximum intensity projection at 100X magnification showing absence of *MYC* (tag present at the N-terminus of both BCR-ABL ^P210^ and BCR-ABL ^T315I^ flies) staining in GFP-positive control hemocytes (a, a’), *MYC* expression in GFP-positive hemocytes indicating expression of *BCR-ABL* ^P210^ (b, b’) and *BCR-ABL* ^T315I^ (c, c’). Scale bar is 10 µm. (B) A significant increase in hemocyte count in *BCR-ABL ^P210^* and *BCR-ABL ^T315I^* expressing larval bleed when compared to control flies and to each other. (C, D) Sketch Diagram and representative images of disruption in sessile hemocyte patterning in *BCR-ABL ^P210^* and *BCR-ABL ^T315I^* flies. Green fluorescence indicates GFP expressed in lymph gland and circulating hemocytes under *Hml Δ-Gal4; UAS-GFP* driver. Regular banding of sessile patterning of hemocytes in third-instar control larva (d), partial disruption of sessile hemocyte in *BCR-ABL* ^P210^ third-instar larva (d’), and complete disrupted patterning of sessile hemocytes in *BCR-ABL* ^T315I^ third-instar larva (d”). (E) Enumeration of banded, partially disrupted, and disrupted patterning in control, *BCR-ABL* ^P210^ and *BCR-ABL* ^T315I^ larva demonstrating the disruptive phenotype in *BCR-ABL* ^P210^ and *BCR-ABL* ^T315I^ larva with a significant exacerbated phenotype in the latter. (F) Analysis in larvae: *Drosomycin, Diptericin A* , and *TotA* relative expressions normalized to *RP49* in control, *BCR-ABL* ^P210^ and *BCR-ABL* ^T315I^ larva hemolymph. A significant increase of all expressions in *BCR-ABL* ^P210^ and *BCR-ABL* ^T315I^ larva when compared to control flies except *Diptericin* *A* which is under-expressed in *BCR-ABL* ^P210^ larva. A significant increase of *Diptericin A* and *TotA* in *BCR-ABL* ^T315I^ larva when compared to *BCR-ABL* ^P210^ larva. (G) Analysis in adults: *Drosomycin, Diptericin A* , and *TotA* relative expressions normalized to *RPL11* in control, *BCR-ABL* ^P210^ and *BCR-ABL* ^T315I^ adult flies. A significant decrease in *Drosomycin* expression in *BCR-ABL* ^T315I^ adult flies when compared to control flies and *BCR-ABL* ^P210^ . *Diptericin A* is overexpressed in *BCR-ABL* ^P210^ adult flies compared to control adult flies. *TotA* is overexpressed in *BCR-ABL* ^T315I^ adult flies compared to *BCR-ABL* ^P210^ and control adult flies. Error bars represent S.E.M B,D,E: n = 30 triplicates tested. F: n = 50 triplicates tested. G: n= 25 triplicates tested. One-way ANOVA with Tukey’s posthoc test (B), Chi-square test (E), and unpaired T-test (F,G) were performed to test statistical significance (*, *p<0.05;* **, p <0.01; ***, p<0.001).

## Description

Chronic Myeloid Leukemia (CML) is a myeloproliferative neoplasm resulting from the BCR-ABL fusion protein coded by the Philadelphia chromosome which results from a reciprocal translocation between the *Abelson* murine leukemia gene ( *ABL* ) on chromosome 9 and *Breakpoint Cluster Region* gene ( *BCR* ) on chromosome 22 (Jabbour and Kantarjian 2018, Nowell and Hungerford 1960, Rowley 1973). This translocation creates the *BCR-ABL* oncogene which codes for the BCR-ABL fusion protein; a constitutively active tyrosine kinase (Lugo et al. 1990). Tyrosine kinase inhibitors (TKIs) are considered the gold standard treatment for CML patients (Hochhaus et al. 2008). Despite the advancement offered by the first and second-generation TKIs, there are certain resistant BCR-ABL mutant leukemic clones. For instance, the T315I mutation which results from the substitution of Threonine with Isoleucine at the 315 ^th^ position of ABL imparts resistance to first and second generation TKIs (O'Hare et al. 2011, Barouch-Bentov and Sauer 2011). One TKI that showed efficacy against the T315I mutation is ponatinib (Tanaka et al. 2010, Chan et al. 2012). However, due to its pan-activity on different kinases, ponatinib has severe side effects and high toxicity and is now administered to patients with precaution (Miller et al. 2014). Despite the extensive research on BCR-ABL, the molecular mechanisms and functional interactors in wild-type P210 and T315I mutant background are yet to be fully understood. Conservation between *Drosophila melanogaster* and mammalian genes (Klein 1997), in addition to the shared homology in proteins that interact with BCR-ABL (Fogerty et al. 1999, Bashaw et al. 2000), suggest that a fly model can be used to understand BCR-ABL genetic interactors (Lo Iacono et al. 2021). We previously validated a CML *Drosophila* model for drug screening using TKIs. We expressed *BCR-ABL* ^P210^ and *BCR-ABL* ^T315I^ in *Drosophila* eyes and observed a rough eye phenotype which was more severe in T315I mutants. Furthermore, we used this eye model to test the efficacy of clinically administered TKIs (Al Outa et al. 2020). Bernardoni *et al.* showed similar rough eye phenotypes upon expression of *BCR-ABL* ^P210^ through interaction with endogenous *Drosophila Abl (Ena)* indicating a conserved signal transduction pathway between humans and flies. In addition, BCR-ABL expression in the hematopoietic precursor cells of the lymph gland affected *Drosophila* blood cell homeostasis by increasing the number of circulating blood cells (Bernardoni et al. 2019). Nevertheless, the impact of BCR-ABL oncogene on innate immunity is yet to be understood. Fruit flies possess two forms of innate immune responses, cellular and humoral. The cellular response is mediated by *Drosophila* hemocytes which can be either circulating or sessile. Circulating hemocytes are present in the hemolymph while sessile hemocytes are found in pockets between the epidermis and muscular layers in the larvae (Shrestha and Gateff 1982, Lanot et al. 2001). Circulating hemocytes can be of three types, macrophage-like plasmatocytes, crystal cells, and lamellocytes. Plasmatocytes phagocytose bacteria and apoptotic debris while crystal cells are involved in the melanization process; the latter is analogous to wound healing. Lamellocytes, the largest in size among circulating hemocytes, are very low in number and differentiate only upon parasitic infection (Rizki and Rizki 1980, Franc et al. 1996). The humoral response on the other hand results in the synthesis of antimicrobial peptides (AMPs) and hemolymph coagulation and melanization. Humoral pathways help *Drosophila melanogaster* discriminate between pathogens based on their surface molecules such that, either one of the NF-κB transcription factors can be activated; Dorsal/Dif or Relish, which are representative of the Toll or IMD pathway, respectively. The JAK/STAT pathway on the other hand was shown to be involved in viral infection whereby Domeless activates the transcription factors Hopscotch and STAT92E, consequently activating the expression of immune and stress-responsive genes such as *Tep1* and *Tot A* (Lemaitre et al. 1997, Lemaitre and Hoffmann 2007). Therefore, the *Drosophila’s* reductionist hematopoietic and immune system would aid in understanding the role of *BCR-ABL* expression on the immune response and hematopoiesis in general.

First, we validated the expression of *BCR-ABL* ^P210^ and *BCR-ABL* ^T315I^ in the hemocytes. We utilized the UAS-Gal4 system to drive oncogene expression in circulating hemocytes and the cortical zone of the lymph gland using *Hml Δ-Gal4;UAS-GFP* (Sinenko and Mathey-Prevot 2004). Upon larval bleed, the expression of GFP was visualized in hemocytes **(Figure 1A a,b,c)** . Control hemocytes lacking transgene expression **(Figure 1A a,a’)** whilst the expression of the *BCR-ABL* ^P210^ **(Figure 1A b,b’)** and *BCR-ABL* ^T315I^ **(Figure 1A c,c’)** were visualized in most of the GFP expressing hemocytes merged images ( **Figure 1A b,c** )

Next, we assessed the effect of expression of both oncogenes on circulating hemocytes (n=30 triplicates). Expression of both *BCR-ABL* ^P210^ and *BCR-ABL* ^T315I^ in hemocytes resulted in a significant increase in circulating hemocyte number compared to control. The average number of circulating hemocytes in control flies was 2.7× 10 ^5^ cells/ml, compared to 9.9 × 10 ^5^ cells/ml in *BCR-ABL* ^P210^ and 13.4 × 10 ^5^ cells/ml in *BCR-ABL* ^T315I^ expressing flies. Notably, *BCR-ABL* ^T315I^ exhibited a significantly higher hemocyte number compared to *BCR-ABL* ^P210^ **(Figure 1B)** .

Furthermore, we assessed sessile hemocyte mobilization since it is one of the key indicators of the immune burden in *Drosophila melanogaster* (n=30 triplicates) (Zettervall et al. 2004, Márkus et al. 2009). This is phenotypically visualized in the banding pattern of the sessile hemocytes in third-instar larvae. Three phenotypic criteria were used to categorize the degree of sessile hemocyte disruption; banded, partially disrupted, and disrupted **(Figure 1C, D)** . The expression of *BCR-ABL* ^P210^ and *BCR-ABL* ^T315I^ resulted in disruption of sessile hemocyte patterning. All control larvae showed normal sessile patterning (100%). The patterning was disrupted in *BCR-ABL* ^P210^ ; with 40% exhibiting partial banding and 40% showing complete disruption. Interestingly, 90% of the *BCR-ABL* ^T315I^ larvae showed complete disruption with merely 10% with partial banding. The increased disruption in *BCR-ABL* ^T315I^ sessile patterning was highly significant compared to *BCR-ABL* ^P210^ and was consistent with the higher increase in circulating hemocytes **(Figure 1E)** .

Given the phenotypic defects observed in the cellular arm of innate immunity in *BCR-ABL* ^P210^ and *BCR-ABL* ^T315I^ expressing larvae, we further examined the status of humoral Toll, ImD, and JAK/STAT pathways at the mRNA level by quantifying downstream effectors such as *Drosomycin, Diptericin A* and *Turandot A* ( *TotA)* at the 3 ^rd^ -instar larval (n=50 triplicates) and adult stages (n=25 triplicates). At the 3 ^rd^ -instar larval stage, expression of *BCR-ABL* ^P210^ and *BCR-ABL* ^T315I^ resulted in dysregulation of all pathways. There was a significant increase in *Drosomycin* and *TotA* expression in *BCR-ABL* ^P210^ and *BCR-ABL* ^T315I^ larva when compared to the control; a mean of 4 and 5-fold increase in *Drosomycin* and a mean of 15 and 30-fold increase in *TotA* expression in *BCR-ABL* ^P210^ and *BCR-ABL* ^T315I^ larva, respectively. Notably, the overexpression of *TotA* in *BCR-ABL* ^T315I^ larva was significant when compared to that of *BCR-ABL* ^P210^ larva. As for *Diptericin A* , a significant under-expression in *BCR-ABL* ^P210^ larva was detected while *BCR-ABL* ^T315I^ larva had a mean of 2-fold increase when compared to the control **(Figure 1F).** Likewise, in the adult stage, the expression of *BCR-ABL ^T315I^* resulted in dysregulation of all pathways. We detected a significant downregulation in *Drosomycin* expression in *BCR-ABL ^T315I^* flies compared to control. However, there was no significant difference in *Drosomycin* expression when comparing BCR *-ABL* ^P210^ to control. In addition, there was a 2.5-fold increase in *Diptericin A* expression in *BCR-ABL ^P210^* flies when compared to the control flies. Furthermore, there was a significant difference in *TotA* relative expression between control flies and *BCR-ABL* ^T315I^ . *BCR-ABL ^T315I^* expressing flies showed a 66.7-fold increase compared to *BCR-ABL* ^P210^ which showed a 5.1-fold increase compared to control ( **Figure 1G** ).

Utilizing an established *Drosophila melanogaster* CML model (Bernardoni et al. 2019), this study demonstrated transformative phenotypes in the hemolymph upon the expression of BCR *-ABL* ^P210^ and *BCR-ABL* ^T315I^ which were represented by an increase in hemocyte count and sessile hemocyte patterning disruption when compared to control flies. Moreover, these phenotypic defects in the cellular arm of innate immunity were associated with a dysregulation in the humoral Toll, ImD, and JAK/STAT pathways at the mRNA level in both the 3 ^rd^ instar larval and adult stages. Interestingly, *BCR-ABL* ^T315I^ flies presented more severe phenotypes and a higher deviation in humoral dysregulation than BCR *-ABL* ^P210^ flies when compared to the control; there were significant differences even between BCR *-ABL* ^P210^ and *BCR-ABL* ^T315I^ flies.

The higher number of hemocytes exhibited in *BCR-ABL* ^T315I^ flies when compared to wild-type *BCR-ABL* ^P210^ flies indicates a more prominent effect of the *BCR-ABL* ^T315I^ mutant on hemocyte homeostasis. The importance of this finding stems from the role hemocytes play as a primary line of defense in *Drosophila* *melanogaster* . A similar increase in the hemocyte number was observed in another study when *BCR-ABL* ^P210^ was expressed in the lymph gland (Baril et al. 2017). Furthermore, since the presence of a stressor such as the expression of oncogenes might result in the recruitment of hemocytes from sessile compartments to the hemolymph (Zettervall et al. 2004), we examined whether *BCR-ABL* expression presents an immune burden and if *BCR-ABL* ^P210^ mobilizes the sessile hemocytes differently from *BCR-ABL* ^T315I^ . We observed more prominent sessile patterning disruption in *BCR-ABL* ^T315I^ . This could highlight differential interaction of *BCR-ABL* ^T315I^ with the extracellular matrix (ECM) that controls hemocyte migration. For instance, Kumar *et al.* , suggested that *BCR-ABL ^T315I^* varies from wild-type *BCR-ABL ^P210^* in its interaction with the ECM. Particularly, the integrin β3/ILK-mediated signaling pathway affects leukemia progression in *BCR-ABL ^T315I^* distinctly compared to wild-type *BCR-ABL ^P210^* . *BCR-ABL ^T315I^* showed lower phosphorylation of focal adhesion kinase (FAK) and integrin-linked kinase (ILK) as well as lower disposition of Fibronectin (Kumar et al. 2020). Furthermore, Moreira *et al* . showed that *zyxin* knockdown (involved in the formation of adhesion sites that connect the ECM to the cellular cytoskeleton) resulted in a significant increase in cell speed and migration (Moreira et al. 2013). Therefore, one possible explanation for the severe disruption in sessile patterning in *BCR-ABL ^T315I^* could be its involvement in activating integrins and adhesion complexes which are required for migration of the sessile hemocytes.

The effect of BCR-ABL on the cellular immunity consequently directed us to assess the status of humoral immune pathways upon oncogene expression. The Toll pathway, for example, is an important regulator of hemocyte number in the hemolymph and their proliferation in the lymph gland (Lemaitre and Hoffmann 2007, Qiu et al. 1998). Toll pathway activation results in *Drosomycin* and *Metchikowin* activation (Hoffmann 2003, Hultmark 2003). When assessing the Toll pathway, there was a significant upregulation in *Drosomycin* transcript level in BCR *-ABL* ^P210^ and *BCR-ABL* ^T315I^ flies during 3 ^rd^ instar larval stage which is maintained in adult flies of the former genotype while the latter is significantly downregulated in the adult stage. Meanwhile, concerning the ImD pathway, *Diptericin A* levels are usually first observed at the 3 ^rd^ instar larval stage which peak at the pupal stage only to decrease once again at the adult stage to levels lower than that of 3 ^rd^ instar larva (Graveley et al. 2011). This pattern was observed in the *BCR-ABL* ^T315I^ flies whereby they had a significantly higher expression at the 3 ^rd^ instar larval stage when compared to the control, only to return to comparable levels once more at the adult stage. However, interestingly BCR *-ABL* ^P210^ flies shifted from having a significant under-expression at the 3 ^rd^ instar larval stage to a significant overexpression at the adult stage when compared to control flies.

As for the JAK/STAT pathway, the *TotA* gene is expressed during the third-instar larval stage, maintained throughout the entire pupal period with very low levels of expression in adult stages only to increase gradually once more 3-4 weeks after eclosure. However, it is induced in adults under stressful conditions such as high temperatures and bacterial challenges (Ekengren and Hultmark 2001, Ekengren et al. 2001). As such, we anticipated an increase in *TotA* expression in all flies at 29ºC. Interestingly, there was a significant increase in *TotA* expression in *BCR-ABL* ^T315I^ flies which was maintained from the 3 ^rd^ instar larval stage into the adult stage while BCR *-ABL* ^P210^ flies demonstrated an increased expression of *TotA* only in the 3 ^rd^ instar larval stage and levels comparable to control flies in the adult stage. This suggests that the *BCR-ABL* ^T315I^ mutant is more stress-tolerant. This may be associated with an increased expression of heat shock proteins, stress-induced MAP kinase cascades, NF-κB signal transduction, and/or JAK/STAT pathway signaling events; all of which could contribute to resistance in CML patients with this mutation against TKIs. In fact, the JAK/ STAT pathway has been studied extensively in CML. STAT5 was shown to maintain the survival and growth of CML cells and JAK2 has been correlated with increased LSC persistence in a TKI background (Warsch et al 2013). Therefore, any of these stress-response signaling elements could be therapeutic targets for a combined therapy with current FDA-approved TKIs. In fact, clinical trials utilizing pegylated interferon (IFN) in combination with second generation TKIs are already underway (Patel et al. 2017). Lastly, due to the significant increase in JAK/STAT pathway in *BCR-ABL* ^T315I^ , reflected in the increase of *Tot A* maintained from 3 ^rd^ instar larval to adult stage *,* associated with the significant decrease in *Drosomycin* during the adult stage echoed the findings by Kim *et al.* which showed that overexpression of the JAK/STAT pathway could decrease *Drosomycin* via the Jra/Stat92E/Dsp1 repressosome complex (Kim et al. 2007). As such, it would be interesting to conduct bacterial challenge assays examining the JAK/STAT pathway, which might elucidate the etiology behind resistance to TKI treatment experienced by CML patients with mutant BCR *-ABL ^T315I^* and whether this strong stress response perpetuated by the JAK-STAT pathway may result in suppression of other critical immune pathways which might affect response to treatment.

## Methods

**Fly stocks**

Fly stocks used were *w1118 (wt)* (BDSC #3605), Hml Δ-Gal4; UAS-GFP (BDSC #30140), *UAS-BCR-ABL ^P210^* (p210) (Al Outa et al. 2020) and *UAS-BCR-ABL ^T315I^ (T315I)* (Al Outa et al. 2020). BCR-ABL1 ^p210^ and BCR-ABL1 ^p210/T315I^ were inserted into pUAST-attB *Drosophila* expression vector (Custom DNA cloning). 1x Myc tag (MEQKLISEEDL) with its own start codon was added at the N-terminus before the transcription start site of BCR (EcorI-and combined speI-xbaI sites). pUAST-attB-myc BCR-ABL1 ^p210^ and pUAST-attB-myc BCR-ABL1 ^p210/T315I^ were injected into y1 w67c23; P {CaryP} ABLattP2 (8622 BDSC) embryos to generate transgenic flies (BestGene Inc, Chino Hills, CA). Fly crosses were performed at 29˚C.

**Immunofluorescence**

For hemocyte staining, late wandering third instar were bled in 10 µl of 1X-PBS in a 12 well plate using a modified protocol (Evans et al. 2014). Then the bleed was transferred to slides and left to attach for 30 minutes in a humidified chamber. The bleed was washed with 1X PBS- 0.3% Triton (PBST) twice for 5 minutes, samples were then blocked in 5% Normal Goat Serum (NGS) in PBST (Dako, Santa Clara, CA) for 2 hours. Anti-Myc antibody (9E-10 kind gift from Bengt Hallberg) 1:300 was used on hemocytes overnight. Samples were washed with PBST 2x5 minutes. Samples were incubated with secondary antibody Alexa-594 1:500 in 5%NGS PBST (Abcam, Cambridge, UK). Samples were washed with PBST 2x5 minutes. Then Fluor shield Mounting Medium with DAPI (Abcam) was added and samples were then imaged using a laser scanning confocal microscope (Carl Zeiss Laser Scanning Microscopy 710, Jena, Germany).

**Hemocyte bleed and count**

Late wandering third-instar larvae were picked from the walls of the food vial with a pair of forceps and cleaned from food and debris by placing them in a 1X PBS containing petri plate before being transferred to a tissue paper to dry them. The larvae were kept in the petri plate on ice throughout the bleed process to minimize their movement. The larvae were placed in 13 µl of 1X-PBS that was placed on a parafilm strip under a light microscope. Using two pairs of forceps the larval cuticle was pierced and hemolymph was released. The larva was left to bleed for 30 seconds. The larva was removed with one side of the forceps so as not to lose a greater volume of the bleed. The hemolymph bleed was mixed thoroughly with a pipette before taking 10 µl of the bleed and placing it in a Neubauer chamber (Buerker-Turk, Marienfeld, Germany) with a coverslip attached. The Neubauer chamber was placed under an Axiostar plus light microscope (Zeiss, Oberkochen, Germany) and the number of cells in each of the four quadrants was noted down. Hemocyte number was then reported as hemocytes per milliliter of bleed. The average number of hemocytes was obtained from three different biological replicates (N=3) with ten third-instar larvae for each replicate. Total number of larvae used in each condition is (n=30).

**Larvae handling and imaging for sessile patterns**

Late wandering third-instar larvae were picked from the walls of the food vial with a pair of forceps and cleaned from food and debris by placing them in a petri plate containing 1X Phosphate Buffered Saline (PBS) (Sigma Aldrich, St. Louis, MO). After drying with a tissue paper, the larvae were placed in an Eppendorf at -80ºC for one and a half minutes. The Eppendorf was then placed on a plate on ice. The larvae were then imaged for sessile patterning using an SZX2-ILLT GFP Olympus microscope (Olympus, Tokyo, Japan) while submerged in a drop of 50% glycerol (Sigma-Aldrich, St. Louis, MO) This is a modified protocol (Anderl et al. 2016). Performed on three different biological replicates (N=3) with thirty third-instar larvae for each replicate. Total number of larvae used in each condition is (n=90).

**RNA extraction**

Third-instar larva:

50 larvae were bled in 20-60 microliter PBS then put in 1 milliliter of Trizol (TRI reagent, Sigma Aldrich). Centrifugated at 12,000g for 15 mins at 4ºC. Then 200 microliters of chloroform (Sigma-Aldrich) were added. Tubes were shaken well then centrifugated at 12,000g for 15 mins at 4ºC. Upper layer was transferred to another Eppendorf and added to it 0.7V of isopropanol 100%. Kept at room temperature for 10mins. Then centrifugated for 30 mins max speed at 4ºC. Isopropanol was removed then RNA pellet was washed with 1 milliliter ethanol 70% (Sigma-Aldrich) which was centrifugated at max speed for 10 mins at 4ºC. Ethanol was removed and Eppendorf was set to air dry for 10 mins under hood. Pellet was resuspended in 20 microliters Nuclease free water (Autoclaved milli-Q water). Performed on three different biological replicates (N=3) with fifty third-instar larvae for each replicate. Total number of larvae used in each condition is (n=150).

Adult flies:

Total RNA was extracted using Trizol from 25 adult flies (3 days old). After adding 150µl of Trizol, flies were ground using a pestle while working on ice. The pestle was washed with additional 150 µl of Trizol. The samples were left to incubate for 5 minutes at room temperature under the fume hood then centrifuged at (14,000 g) at 4˚C for 5 minutes. All centrifugations were done at 4˚C at maximum speed (14,000 g). Chloroform was added to the Trizol in a ratio of 1:5 (Chloroform: Trizol). The samples were mixed and incubated for 1 minute and centrifuged for 10 minutes. The upper layer was transferred, and isopropanol was added. The mixture was incubated at room temperature for 10 minutes and then centrifuged for 30 minutes. Samples were then washed with 70% ethanol twice and centrifuged for 10 minutes. the pellet was air-dried and resuspended in 30 µl Nuclease free water. Performed on three different biological replicates (N=3) with 25 adult flies for each replicate. Total number of larvae used in each condition is (n=75).

**Quantitative PCR**

Real time PCR was performed using SYBR green (Bio-Rad). Each sample was run in triplicates. Bio-RAD CFX96 Real time system was used for real time analysis. Relative gene expression was analyzed using the Livak and Schmittegn system (Livak and Schmittgen 2001). The assessment was repeated for three biological replicates where each biological group included 50 third-instar larva/25 adult flies. In the case of the larva, expression levels were normalized against the housekeeping gene *Ribosomal protein 49* ( *Rp49* ). While in the case of the adult flies, expression levels were normalized against the housekeeping gene *Ribosomal protein L11* ( *RpL11* ). The Forward and Reverse primers (Macrogen, Seoul, South Korea) sequences used all have an annealing temperature of 57 °C. Sequences are reported in the Reagents section. Targets tested were *Drosomycin, Diptericin A,* and *TotA.*

**Statistical Analysis Results**

These statistical analyses were performed on a GraphPad Prism 6.0 program. P-values lower than 0.05 were considered statistically significant.

**Table:** 

**Figure Panels**	**Statistical Test**		**P-value**
B	ANOVA		<0.0001
B	Tukey’s post-hoc test	wt vs. BCR-ABL ^P210^	<0.0001
B	Tukey’s post-hoc test	wt vs. BCR-ABL ^T315I^	<0.0001
B	Tukey’s post-hoc test	BCR-ABL ^P210^ vs. BCR-ABL ^T315I^	<0.0001
E	Chi-square test	Banded: wt vs. BCR-ABL ^P210^	<0.0001
E	Chi-square test	Banded: wt vs. BCR-ABL ^T315I^	<0.0001
E	Chi-square test	Banded: BCR-ABL ^P210^ vs. BCR-ABL ^T315I^	<0.001
E	Chi-square test	Partially: wt vs. BCR-ABL ^P210^	<0.0001
E	Chi-square test	Partially: wt vs. BCR-ABL ^T315I^	0.0115
E	Chi-square test	Partially: BCR-ABL ^P210^ vs. BCR-ABL ^T315I^	0.0018
E	Chi-square test	Disrupted: wt vs. BCR-ABL ^P210^	<0.0001
E	Chi-square test	Disrupted: wt vs. BCR-ABL ^T315I^	<0.0001
E	Chi-square test	Disrupted: BCR-ABL ^P210^ vs. BCR-ABL ^T315I^	<0.0001
F	Unpaired T-test	*Drosomycin* : wt vs. BCR-ABL ^P210^	<0.001
F	Unpaired T-test	*Drosomycin:* wt vs. BCR-ABL ^T315I^	<0.001
F	Unpaired T-test	*Drosomycin:* BCR-ABL ^P210^ vs. BCR-ABL ^T315I^	0.47
F	Unpaired T-test	*Diptericin A:* wt vs. BCR-ABL ^P210^	<0.001
F	Unpaired T-test	*Diptericin A:* wt vs. BCR-ABL ^T315I^	<0.001
F	Unpaired T-test	*Diptericin A:* BCR-ABL ^P210^ vs. BCR-ABL ^T315I^	<0.001
F	Unpaired T-test	*TotA:* wt vs. BCR-ABL ^P210^	<0.001
F	Unpaired T-test	*TotA:* wt vs. BCR-ABL ^T315I^	<0.001
F	Unpaired T-test	*TotA:* BCR-ABL ^P210^ vs. BCR-ABL ^T315I^	<0.001
G	Unpaired T-test	*Drosomycin* : wt vs. BCR-ABL ^P210^	0.47
G	Unpaired T-test	*Drosomycin:* wt vs. BCR-ABL ^T315I^	0.0068
G	Unpaired T-test	*Drosomycin:* BCR-ABL ^P210^ vs. BCR-ABL ^T315I^	0.0365
G	Unpaired T-test	*Diptericin A:* wt vs. BCR-ABL ^P210^	0.0442
G	Unpaired T-test	*Diptericin A:* wt vs. BCR-ABL ^T315I^	0.9008
G	Unpaired T-test	*Diptericin A:* BCR-ABL ^P210^ vs. BCR-ABL ^T315I^	0.0713
G	Unpaired T-test	*TotA:* wt vs. BCR-ABL ^P210^	0.0578
G	Unpaired T-test	*TotA:* wt vs. BCR-ABL ^T315I^	0.0022
G	Unpaired T-test	*TotA:* BCR-ABL ^P210^ vs. BCR-ABL ^T315I^	0.0030

## Reagents

Table 1: Drosophila melanogaster stocks used:

**Table:** 

	**Genotype**	**Source**	**Stock #**
*w1118 (wt)*	w[1118]	Bloomington *Drosophila* Stock Center	3605
Hml Δ-Gal4; UAS-GFP	w[1118]; P{w[+mC]=Hml-GAL4.Delta}2, P{w[+mC]=UAS-2xEGFP}AH2	Bloomington *Drosophila* Stock Center	30140
* UAS-BCR-ABL ^P210^*	* UAS-BCR-ABL ^P210^*	(Al Outa et al. 2020).	FBtp0141454
* UAS-BCR-ABL ^T315I^*	* UAS-BCR-ABL ^T315I^*	(Al Outa et al. 2020).	FBtp0141455

Table 2: Primers used for q-PCR:

**Table:** 

Target Gene	Primer	Sequence (5’ to 3’)
*Drosomycin*	Forward	TACTTGTTCGCCCTCTTCG
	Reverse	GTATCTTCCGGACAGGCAGT
*Diptericin A*	Forward	CCGCAGTACCCACTCAATCT
	Reverse	ACTGCAAAGCCAAAACCATC
*TotA*	Forward	CCCAGTTTGACCCCTGAG
	Reverse	GCCCTTCACACCTGGAGA
*RP49*	Forward	CGGATCGATATGCTAAGCTGT
	Reverse	GCGCTTGTTCGATCCGTA
*RPL11*	Forward	CGATCCCTCCATCGGTATCT
	Reverse	GCCCTTCACACCTGGAGA
